# Secular Trends in Head Size and Cerebral Volumes In the Framingham Heart Study for Birth Years 1902–1985

**DOI:** 10.21203/rs.3.rs-2524684/v1

**Published:** 2023-01-30

**Authors:** Charles DeCarli, Matthew Pase, Alexa Beiser, Daniel Kojis, Claudia Satizabal, Jayandra Himali, Hugo Aparicio, Evan Flether, Pauline Maillard, Sudha Seshadri

**Affiliations:** University of California, Davis; Monash University; Department of Biostatistics, Boston University School of Public Health; Boston University; Glenn Biggs Institute for Alzheimer’s and Neurodegenerative Diseases and Department of Population Health Sciences, UT Health San Antonio, San Antonio, TX; Glenn Biggs Institute for Alzheimer’s and Neurodegenerative Diseases and Department of Population Health Sciences, UT Health San Antonio, San Antonio, TX, USA; Boston University; University of California, Davis; University of California, Davis; University of Texas Health Science Center

## Abstract

Background Recent data suggest that dementia incidence is declining. We investigated whether similar secular trends consisting of increasing size of brain structures and improving memory performance could be simultaneously occurring as a possible explanation. Method The Framingham Heart Study is a 3 generation, longitudinal study that includes cognitive assessment and medical surveillance. This study cohort consisted of 4,506 unique, non-demented, stroke free, individuals with brain MRI, cognitive assessment, and demographic information spanning dates of birth from 1902 to 1985. Outcomes consisted of height, MRI, and memory measures. Covariates included age at MRI, sex, decade of birth, and all interactions. Models with neuropsychological outcomes also included educational achievement as a covariate. Results Height and intracranial (TCV), hippocampus and cortical gray matter volumes were significantly larger, and memory performance significantly better, with advancing decades of birth after adjusting for age, sex, and interactions. Sensitivity analysis using progressively restricted age-ranges to reduce the association between age and decade of birth, confirmed the findings. Mediation analysis showed that hippocampal volume mediated approximately 5–7% of the effect of decade of birth on logical memory performance. Discussion These findings indicate improvement in brain health and memory performance with advancing decades of birth. Although brain structures are under substantial genetic influence, we conclude that improved early life environmental influences over ensuing decades likely explain these results. We hypothesize that these secular improvements are consistent with declining dementia incidence in this cohort potentially through a mechanism of increased brain reserve.

## Introduction

The overall health of the U.S. population has improved dramatically over the last 100 years. Infant mortality has declined precipitously due to improved sanitation, nutrition, and medical treatment^1^ although health disparities persist^2^. Individuals are also living longer resulting in an increasing percentage of the population at risk for Alzheimer’s disease and related dementias (ADRD). Recent data^3, 4^, including from the Framingham Heart study^5^, however, finds that dementia incidence may be declining. While many factors such as greater educational achievement^6^ and medical management of vascular risk factors^7, 8^ may explain part of this effect, early life environmental differences likely also contribute^6^.

The Framingham Heart Study, initiated in 1948, consists of multiple generations of participants across more than 80 years difference in birth dates, making it ideal for investigating secular trends in cardiovascular and brain health^7, 9^. The Framingham MRI study, initiated in 1999, has imaged 5,145 unique individuals across all three birth cohorts enabling age-specific comparisons of secular trends. We hypothesized that secular trends in improving early-life health, as observed in the general US population, would be accompanied by the expected increase in height as well as head and brain size. Given that increasing head size has been shown to be independently associated with improved later-life cognitive performance^10^ we further hypothesized that these secular trends would significantly associate with similar trends in improved levels of education and cognitive ability, and that increased hippocampal volume would partially mediate the impact of decade of birth on memory performance indicating a possible mechanism of brain reserve that could explain declining dementia incidence in this cohort.

## Methods

### Study Design

The general design and demographics of the Framingham Heart Study (FHS) have been previously described^9, 11, 12^. In brief, the Framingham Heart Study is a community-based population study of over 15,000 individuals from the town of Framingham Massachusetts, USA spanning more than 75 years of observation. The Original cohort of the Framingham Heart Study included 5,209 participants who were enrolled into the study in 1948. At enrollment, the mean age was 44 years (range 28–62 years) and 55 per cent were female, the majority of whom were white and of middle socioeconomic class. Surviving members of this cohort are examined approximately every two years. The Offspring cohort included 5,124 offspring of the original cohort and their spouses enrolled in 1971 with examinations performed approximately every 4 years. At enrollment, the mean age was 36 years (range 25–70 years) and 52 percent were female. The 3rd generation cohort included 4,095 children of the Offspring cohort enrolled in 2002 and has completed 3 evaluations to date. At enrollment, the mean age was 41 (range 25–70 years) and 53 percent were female. In addition to repeated examination cycles, surviving participants are under constant surveillance for incident events such as myocardial infarction, stroke, and dementia as part of regular health evaluations that included assessment of vascular risk factors (e.g. Framingham Stroke Risk Profile [FSRP]^13^, systolic blood pressure [SBP], diabetes, etc.). As part of multiple large ancillary studies on brain structure and cognitive function, FHS participants were recruited, starting in March 1999, to undergo MRI of the brain and to be administered an neuropsychological test battery as previously described^14^.

**Figure 1:**
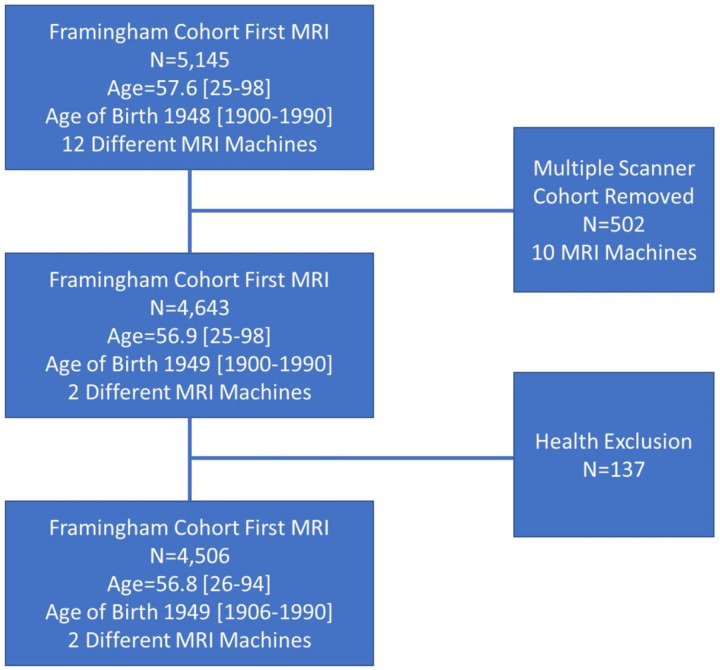
Sample Selection Figure 1 summarizes subject selection with mean age and standard deviation. All individuals were included at the time of the first MRI. Subjects were imaged on a total of 12 different MR machines, but greater than 90% of participants were imaged on only 2 different scanners leading to removal of 502 participants from study due to imaging on alternative scanners. Finally, individuals with stroke, dementia of other neurological disorder were excluded resulting a study cohort of 4506 individuals.

To examine secular trends in MRI and cognitive performance, initial visits where MRI and neuropsychological testing were performed were selected. This resulted in a cohort of 5,145 individuals with a mean age of 58 (range 25–98 years) who were born between 1900 and 1990. MRI was performed between the years 3/18/1999 and 11/15/2019 on a total of 12 different MRI machines. Most participants, however, were scanned on only two different Siemens machines and to reduce the impact of machine variability on MRI measures, this subgroup of 4,643 individuals were selected for this study. Of the 4,643 participants, 137 were excluded due to prevalent dementia, stroke, or other Significant neurological disorder (e.g., multiple sclerosis) at the time of MRI, reducing the final number for analysis to 4,506 (see Fig. 1). All participants have provided written informed consent prior to study. Study protocols were approved by the institutional review board at Boston University Medical Center.

### Anatomical outcomes

#### Midlife height

Height measurement in inches was obtained at the first FHS visit for each participant and was made at an average age of 35, 36 and 41 years across the 3 cohorts, and 37.7 ± 8.1 years of age on average, avoiding the impact of later life skeletal degeneration on the measure.

#### Brain Measures

Quantifications of MRI measures were obtained from high resolution 3D T1 weighted MP RAGE images using either a Siemens Magneton Expert or Siemens Avanto machine to obtain intracranial, cortical gray matter, hippocampal volume, and cortical thickness measures. Quantification included automatic removal of non-brain elements from the 3D T1 image volume using a robust and accurate method relatively insensitive to machine type or field strength^15^. For this study, intracranial volume was defined as the cranial vault above the tentorium. Image intensity correction was used to remove B1 inhomogeneity effects^16^ and segmentation of the image into 3 tissue classes used an algorithm optimized to improve precision of tissue segmentation (gray, white, CSF^16, 17^). Additionally, hippocampal analyses were performed using an atlas based diffeomorphic approach^18^ with the minor modification of label refinement. Cortical thicknesses were measured using the DireCT method^19^. Cortical surface area was calculated using 3D cortical surface mesh reconstructions employing marching cubes^20^, a standard algorithm in computer graphics that has been used extensively in brain image analyses^21^. To estimate gray matter surface area in a region of interest in MRI native space, we compute the area of the polygonal facets lying within the ROI mask.

Cortical parcellation used the Desikan-Killiany-Tourville Atlas^22^ where regional measures were calculated by back transformation of the atlas into segmented image native space. A voting scheme is used to assure precise labelling of each region after interpolation of the atlas into native space. Using this approach, we defined four measures for study: 1) Intracranial volume (TCV; in cubic centimeters; cc), 2) Total cortical gray matter volume (cc), 3) Hippocampal volume (cc), 4) Surface area in squared centimeters (cm^2^) and 5) Average cortical thickness in millimeters (mm). To correct for scanner effect, MRI measures were corrected using NeuroComBat, a robust method for reducing machine related differences in MRI data^23–25^ (See Supplemental Materials for details).

#### Neuropsychological outcomes

Neuropsychological assessment consisted of a battery of tests of verbal learning and memory (Wechsler Memory Scale-III [WMS-III] Logical Memory and Paired Associates), visual memory (WMS-III Visual Reproduction), abstract reasoning (Wechsler Adult Intelligence Scale-III [WAIS-III] Similarities, visuospatial skill (Hooper VOT), language (Boston Naming Test) and executive functioning (Trails A and B) as previously described^26^. Given that verbal memory performance is both sensitive to brain aging^27–29^ and predicts future clinically relevant cognitive impairment^30^, analyses for this study focused on verbal memory variables of immediate, delayed and the sum of both immediate and delayed recall.

#### Genetics, Vascular risk factors and education

APOE genotype was determined with a standard TaqMan assay; APOE-e4 carriers were defined as those having at least one e4 allele. The Framingham Stroke Risk Profile (FSRP) score is a validated, widely-used composite measure of vascular risk factors that predicts the 10-year probability of a stroke^31^. Systolic blood pressure (SBP, mmHg) was taken as the average of the Framingham clinic physician’s two measurements. Educational level was defined as the obtainment of a college degree (yes or no).

### Statistical Analysis

#### Height and Head Size

To understand the relationship of decade of birth with body height and cranial volume, we performed univariate associations between mid-life height, intracranial volume, and decade of birth, stratified by sex at birth.

In addition, because there are striking sex-differences in both these features, we investigated possible sex-differences in these relationships using multivariate analyses to test the interaction between mid-life height, intracranial volume, decade of birth and sex at birth. Given that height and cranial volume are correlated, we also performed multivariate analysis to investigate if cranial volume differed by decade of birth adjusting for height.

#### Brain Measures

To understand the relationship of decade of birth with brain measures, we performed multi-variate linear regression analyses of TCV, cortical gray matter, cortical thickness, and hippocampal volumes with decade of birth including sex at birth, age at MRI, and all possible interactions as covariates.

While many brain aging studies correct regional brain volumes for head size to reduce variance, we recognized that any secular differences in head size would influence our results. Consequently, we examined each brain region independently without adjusting for intracranial volume to avoid confounding by secular differences in intracranial volume (TCV).

#### Contributions of ApoE genotype and Vascular Risk Factors

We used logistic and linear regression to examine the association between vascular risk factors, including ApoE genotype (E4 genotype yes/no), FSRP and, SBP with decade of birth including sex at birth, and age at MRI and all possible interactions as covariates. We then examined the influence of these factors on the association between decade of birth and brain measures by adding to the models described above ApoE genotype, FSRP and, SBP as additional covariates, as well as the interaction of these risk factors with decade of birth.

### Sensitivity Analysis

Initial analysis included the full range of decade of birth and age at MRI. Given the likely association between decade of birth and age at MRI, sensitivity analyses were also performed to limit the impact of age at MRI on the observed associations. The first analysis limited the age range at MRI from 45 to 75 years in 3 ten-year epochs. A second analysis further limited the age range at MRI to 55–65 years of age.

### Impact of Decade of Birth on Neuropsychological Outcomes

#### Contribution to Education

Given the known, strong relationship between educational achievement and memory task performance, we first used a logistic regression to examine the association between achieved college education with decade of birth including sex at birth, and age at MRI and all possible interactions as covariates.

#### Contribution to Logical Memory Performance

We then examined the impact of decade of birth on verbal memory performance (Logical Memory, immediate recall, delayed recall, and the sum of both) using multi-variate linear regression analyses with decade of birth, sex at birth, age at MRI, educational achievement, and all possible interactions as covariates.

#### Neuropsychological Association with Brain measures

We further investigated the effect of brain measures on verbal memory performance using linear regressions with decade of birth, sex at birth, age at MRI, educational achievement, and all possible interactions as covariates.

#### Hippocampal Volume Mediating Effect

Given the likelihood that brain structure would increase, and verbal memory performance improve, we examined whether hippocampal volume might mediate the relationship between decade of birth and verbal memory performance. Causal mediation analysis was used to test the mediation effect of hippocampal volume on the relationship between decade of birth and memory performance adjusting for sex at birth, age at MRI, educational achievement, and all possible interactions as covariates using 5,000 bootstrap iterations for estimation.

All MRI measures and neuropsychological test performances were converted to Z-score values for relative comparison of results across measures. Analyses were performed using R version 4.1.2. Mediation analyses used the R Mediation package which is based on methods of Imai et al^31^.

## Results

### Sample Characteristics

The final cohort sample is summarized in Table 1 and consisted of 4506 individuals, of which 54% were female. The average age at MRI was 56.8 ± 12.6 years but ranged from 25 to 94 years. The average decade of birth was the 1940s but ranged from 1910 to 1980. More than three-quarters of the individuals (76%) were college educated and had a relatively low prevalence of cerebrovascular risk factors^32^ as determined by clinical examinations an average of 1.1 ± 0.9 years around the time of MRI. The average time interval between neuropsychological testing and MRI was 0.01 ± 0.09 years and ranged from 0 to 1.33 years with 90% of subjects receiving neuropsychological testing at the time of MRI. Significant differences by sex at birth were found among women for height (−2.74 inches), intracranial volume (−77.9 ccs), hippocampus (−0.32 ccs), total cortical gray matter (−27.0 ccs), immediate memory (0.51 points greater) and delayed verbal memory (0.55 points greater). The specific distributions of age for various decades of birth are also summarized in the Supplemental Materials to this manuscript.

### Impact of Decade of Birth on Anatomical Outcomes

#### Height and Head Size

**Figure 2:**
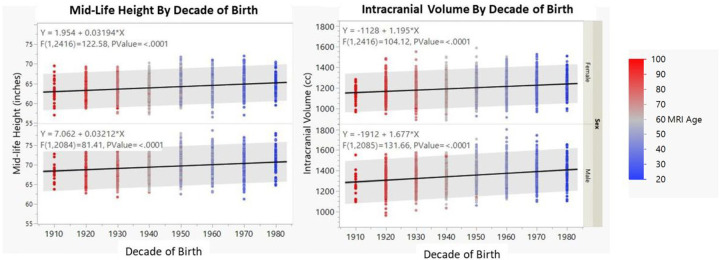
Height and Intracranial Volume Measures by Decade of Birth Figure 2 summarizes the impact of decade of birth on height and intracranial volume (converted to z-scores) for men and women colored by decade of age at time of MRI.

Figure 2 summarizes the findings for height and head size by decade of birth. First, we separately examined differences in height and head size. Height increased significantly over decades of birth for both men and women with nearly an identical slope (0.032 inches per decade). Intracranial volume also increased significantly for both men and women over the decades of birth with modest, but not Significant differences by sex (1.2 cc/decade for women versus 1.7 cc/decade for men). Next, we tested whether the increases in head size differed from those for height. In a multivariate model that included adjustments for height, sex, age, and interactions of sex with age, height and decade of birth, intracranial volume increased slightly but significantly at 1.41 cc per decade of birth, indicating small but Significant secular increases in intracranial volume relative to height for men and women (p < 0.0001).

### Brain Measures

The results of our primary analyses are summarized in Table 2 (leftmost column, for the full range of ages under each brain region) where brain measures were converted to z-scores to compare effects across regions. For all brain measures except cortical thickness, Significant positive effects of decade of birth were found for each region with the magnitude being greatest for intracranial volume. In contrast, decade of birth was significantly and negatively associated with cortical thickness.

**Figure 3:**
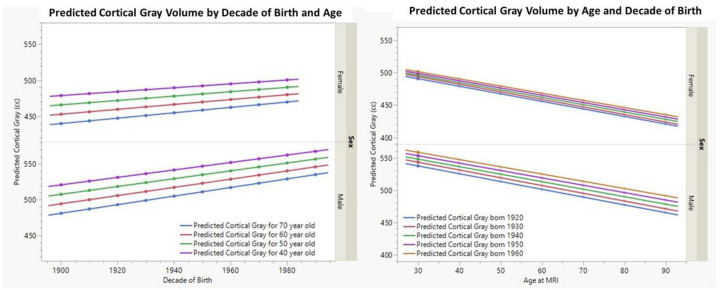
Relationship between Cortical Gray Matter Volume, Decade of Birth and Age Figure 3. Left panel summarizes the predicted impact of decade of birth on cortical gray matter volume (converted to z-scores) for men and women and stratified by decade of age at time of MRI. Increasing age is significantly associated with lower cortical gray matter volume for both men and women whereas increasing decade of birth is associated with increased volume of cortical gray matter for both men and women. The right panel displays the predicted relationship of age to cortical gray matter for men and women stratified by decade of birth. The stronger impact of aging versus decade of birth is clearly seen in this panel.

N denotes numbers of participants at each level of sensitivity analysis. Age-range of restricted analyses is given as heading to that column.^1^ All MRI measures were z-transformed for comparison of effect across regions and results presented as beta coefficients from regression analysis. Figure 3 summarizes associations between total gray matter volume (cc) for men and women of the study by decade of birth (left panel) and age (right panel) predicted from the multiple regression parameters summarized in Table 2. The left panel displays the relation between decade of birth and total gray matter volume by various ages, whereas the right panel displays the relation between age and total gray matter volume by various decades of birth. As an example, from the left panel, a 60-year-old man born in 1920 would be predicted to have a cortical gray matter volume of 501 cc whereas a 60-year-old man born in 1980 would be predicted to have 537 cc of cortical gray matter. From the right panel, a man born in 1948 (average birth decade of the study population) would be predicted to lose nearly 80 ccs of gray matter volume over the same time frame (ages 25–85). Therefore, while decade of birth had a Significant positive effect of cortical gray matter volume, the impact of aging on cortical gray matter remains essentially unchanged (i.e. no Significant age by birth decade interaction) and the standardized beta is greater in magnitude for the age effect versus the impact of decade of birth (β_age(yrs)_ = −0.021 versus β_birth decade_ = 0.008 z-scores), although opposite in sign (i.e. increasing age leads to atrophy whereas increasing birth decade leads to larger volume).

### Contribution of ApoE genotype and Vascular Risk Factors

Logistic regression analysis of the prevalence of ApoE4 genotype by decade of birth was not Significant. Conversely, SPB measures declined significantly with advancing decade of birth (p < 0.0001). Both FSRP and SPB, however, strongly interacted with age and decade of birth (p < 0.0001) in predicting cortical GM volume outcomes, where the positive impact of decade of birth on brain structure was diminished by increased participant age (and consequently worsening FSRP or SPB) at the time of MRI.

Adding ApoE genotype to the MRI models resulted in a very small (0.03 cc), but Significant (p < 0.05) increase in TCV and cortical gray matter volumes for ApoE4 genotype carriers that did not modify the positive effect of decade of birth on these measures. Conversely, FSRP, a summary scale for stroke risk^33^, was associated with significantly smaller, hippocampal, and cortical gray matter volumes (p < 0.0001), that interacted modestly with decade of birth such that declining mean FSRP with increasing decade of birth was associated with increased volumes of these measures.

### Sensitivity Analyses

Given the substantial association between age at time of MRI and decade of birth, two additional analyses, selecting more limited age-ranges, were performed. Table 2 includes the results of our analysis restricting the age range of participants. The number and distribution of ages within these subsamples are summarized in the Supplemental Materials to this study. The sensitivity analysis was done at two levels of restriction. The first step was to reduce the range to 45–75 years of age in epochs of 10 years, spanning the birth decades of 1930 to 1970. These results are shown in the second column of Table 2 under each MRI measure. While age continues to influence brain measures, the impact on decade of birth was not changed in this age-restricted group. In the second sensitivity analysis, the age range was further restricted to a single 10-year epoch of ages between 55 and 65 that spanned the decades of birth from 1940 to 1960. The results are shown in the third column of Table 2 under each MRI measure. Comparing results across the various groups, the age effects diminish as the age ranges are restricted, but the impact of decade of birth is equal to, or increased with each level of analysis, except for cortical thickness where the effect of birth decade diminishes with restricted age range but remains significantly negative.

**Figure 4:**
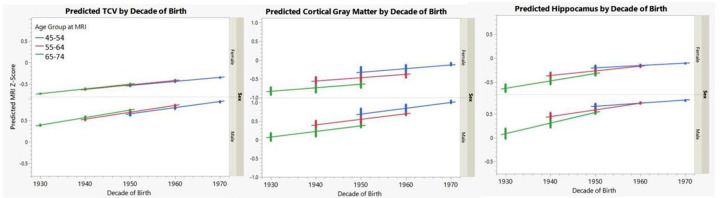
Sensitivity Analyses: Predicted MRI regions by Decade of Birth for Three Age Groups from 45–75 years of Age Figure 4 summarizes the predicted impact of decade of birth on TCV, cortical gray matter and hippocampal volumes (converted to z-scores) by decade of birth for a subset of individuals between the ages of 45 to 75 (3 age decades). Linear increases in TCV are seen for men and women by decade of birth without Significant age effects, whereas decade of birth differences, while steadily increasing also show aging effects with smaller volumes by age decade.

Figure 4 (this figure and all subsequent figures use z-score converted measures to compare across MRI regions and cognitive performance measures examined) summarizes predicted results for those individuals 45–75 years of age at the time of MRI by decade of birth, where color coding denotes age group at MRI. In brief, there is a linear increase in predicted intracranial volume with increasing decade of birth within and across the 3 age-ranges. This is expected given that intracranial volume does not vary significantly with age in this subgroup. Conversely, both cortical gray matter and hippocampal volumes show a Significant age effect, where older individuals have smaller volumes, but again, the volumes increase with advancing birth decade, even within the same age group (as denoted by the same color lines). Given that the age lines are generally parallel, the effects of decade of birth appears linear across the age groups for all MRI measures, except for hippocampal volume, where the effect seems largest for the oldest age group.

**Figure 5:**
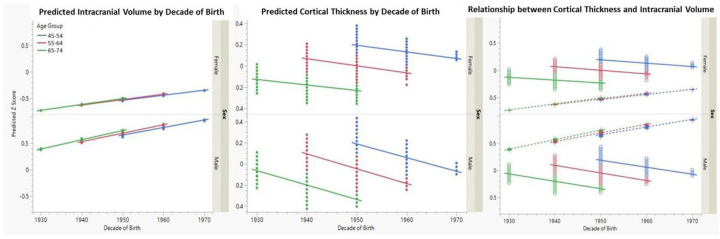
Sensitivity Analyses: Relationship of Decade of Birth and Predicted Intracranial Volume and Cortical Thickness Figure 5 displays a similar relationship with TCV (converted to z-scores) in the left panel, cortical thickness (converted to z-scores) in the middle panel and the co-variation of TCV and cortical thickness in the third panel. TCV increases, whereas cortical thickness decreases with decade of birth as well as advancing age. Overlaying of the two processes, however, indicates that decade of birth increases in TCV is associated with cortical thinning.

The decade of birth relationship is different for cortical thickness as summarized by Fig. 5. In this figure, cortical thickness declines with advancing age, but there is also a decline in cortical thickness with advancing decade of birth. Overlay of intracranial volume (most right panel) shows declines in cortical thickness across birth decades to covary with increasing head size.

To further investigate this finding, we examined the relationship between intracranial volume, cortical surface area and cortical thickness, as cortical surface multiplied by cortical thickness should reflect cortical gray matter volume. For each cc in intracranial volume, there was 0.35 cc increase in gray matter (R^2^ = 0.88, p < 0.0001). Similarly, for each cc in cortical gray matter volume, cortical surface area increased by nearly 3 cm^2^ (R^2^ = 0.63, p < 0.0001) but for each cc in cortical gray matter volume, cortical thickness increased by only 0.001 mm (R^2^ = 0.13, p < 0.0001). Conversely, for each cm^2^ increase in cortical surface area, cortical thickness declined by 0.003 mm (R^2^ = 0.14, p < 0.0001). These results indicate that the increased head size and gray matter accompanying advancing decade of birth led to increases in surface area that were greater per unit increase than cortical thickness, leading to the negative association of cortical thickness with decade of birth within similarly aged individuals.

### Impact of Decade of Birth on Neuropsychological Outcomes

#### Contribution to Educational Achievement

Logistic regression found a highly Significant increase in likelihood of achieving a college education with each advancing decade of birth, reaching a peak probability of nearly 92% for women born in 1970 (p < 0.0001). There also was a Significant 2-way interaction between decade of birth and sex (p < 0.01) where women had more rapidly increasing likelihood of achieving a college education with advancing decade of birth, particularly between decades of birth spanning 1910–1950.

#### Memory Performance

**Figure 6:**
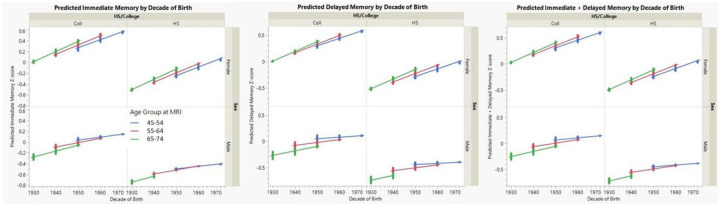
Impact of Decade of Birth and Education on Memory Performance Figure 6 displays the predicted relationship between decade of birth and immediate, delayed and the sum of immediate and delayed memory performance (converted to z-scores) for men and women stratified by level of education (college versus non-college). College education had a profound positive influence on memory performance. Memory performance also improved by decade of birth, although this effect appears strongest for the oldest age cohort.

The impact of decade of birth on memory performance is summarized in Table 3 and displayed for the 45–75-year age groups in Fig. 6. Overall, women performed better than men on these memory tasks. Decade of birth was positively and significantly associated with memory performance on all measures (p < 0.001−< 0.0001). The main effect of age was not Significant, but there was a modest but Significant interaction where older individuals appear to improve more at later birth decades (p < 0.05). An example of this effect is seen in Fig. 6 where the slope of improvement by decade of birth seems greatest for the 65–75-year age group and flattens slightly in the younger age groups. This effect also appears strongest among men. An additional, striking effect of education on memory performance for college educated individuals is also seen and is greatest for women although the 3-way interaction of education*sex*decade of birth (not shown), was not Significant.

#### Contribution of MRI Measures to Memory Performance

Intracranial, cortical gray and hippocampal volumes were significantly associated with each measure of memory performance after adjusting for age, sex, and education (p < 0.001−<0.0001). Cortical thickness was not associated with memory performance. When intracranial, hippocampal, and cortical gray matter volumes were included in the same model, however, only hippocampal volume remained significantly associated with memory performance (p < 0.001−<0.0001). Finally, hippocampal volume remained significantly associated in a model that included the addition of decade of birth with age, sex, and education as predictors of memory performance (p < 0.0001).

#### Mediation Effect of Hippocampal Volume

**Figure 7:**
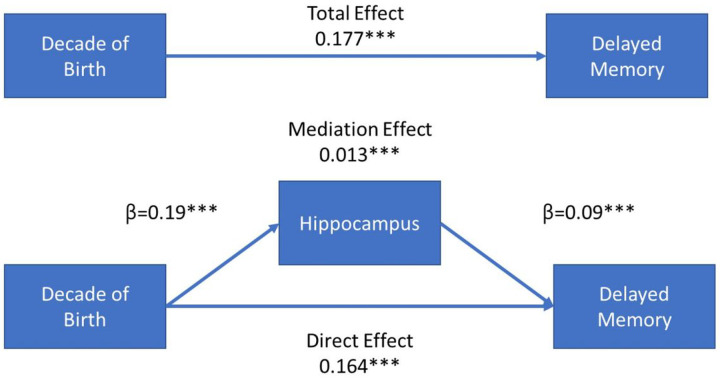
Mediation of Hippocampus between Decade of Birth and Delayed Memory Performance Figure 7 summarizes the mediation effect of hippocampal volume (converted to z-scores) on the relationship between decade of birth and delayed memory performance. The top panel summarizes the direct relation between decade of birth and memory performance. The bottom panel summarizes the mediation effect of hippocampal volume. Hippocampal volume was positively and significantly associated with decade of birth and memory performance and explained approximately 7% of the variance in the relationship between decade of birth and delayed memory performance (mediation effect/direct effect).

We assessed the possible mediation of hippocampal volume on the relationship between decade of birth and memory performance using causal mediation analysis. Table 4 summarizes the results. Hippocampal volume significantly mediated the relationship between decade of birth and memory performance. The proportion mediated by hippocampus varied from approximately 5 to 7%, being strongest for delayed memory mediation. The components of the mediation analysis are summarized in Fig. 7 where hippocampal volume is positively associated with decade of birth and delayed memory performance with an average causal mediation effect of 0.013 (p < 0.0001) for delayed memory.

## Discussion

Our results find Significant secular increases in height and brain structure along with improved memory performance with advancing decades of birth. Brain structures also appear to be increasing at a rate slightly greater than height. Further, improved memory performance accompanying advancing decade of birth appears to be mediated (at least partially) by increasing hippocampal volume after adjusting for age, sex, and education. We hypothesize that larger brain volumes indicate improved “brain health”^34, 35^ and potentially greater “brain reserve”^36–41^ that could explain the declining incidence of dementia as previously reported in the Framingham Heart Study^5^ and further supported by improved verbal memory performance associated with greater hippocampal volume found in this study.

Our findings of consistent enlargement of TCV, cortical gray matter and hippocampal volume with advancing years of birth spanned dates from 1902–1985. Confounding by association of age at MRI with decade of birth was addressed through sensitivity analysis. Results from these analyses found that stepwise restriction of the age of participants, first to 45–74, and then to 55–65 years, where age-related differences in brain volumes were minimal, showed consistent increases in brain size measurements with advancing decade of birth, even when the decades of birth were limited to 30 years as occurred with the most restricted analysis (See Supplemental Materials). Analysis of the age-range between 45 and 75 years of age (equal to the middle quartiles of the age distribution or 75% of the cohort) and covering 5 decades of birth, resulted in multiple unique findings (Figs. 4–6). First, TCV volume, like height, increased linearly through the 5 decades for both men and women. Conversely, decades of birth effect were strongest for hippocampal volume for individuals in the 7th decade of life, and somewhat less for those in the 6th decade, at the time of MRI. These birth years from 1930–1950 coincided with times of great stress such as the depression and World War II. Stress is known to affect hippocampal structure and function^42, 43^ and it is tempting to postulate these major stressors as potential factors contributing to secular trends in hippocampal volume along with the many other differences lifestyle that occurred over the same time period. Finally, cortical thickness declined with decades of birth as well as advancing age. Declining thickness coincided with increasing TCV as well as cortical gray matter volume and surface area. Although cortical surface area was positively associated with cerebral gray matter, cortical surface area was inversely associated with cortical thickness. Rakic^44^ describes how the radial unit lineage model could explain evolutionary and developmental aspects of the cerebral cortex that would be consistent with our findings. In this model, expansion of the cerebral cortex occurs through increasing convolutions and expanding surface area while limiting change in cortical thickness. White et al^45^ extend this concept to “gyrification” of the cerebral cortex, which they note results in a surface area “1700 times larger [in humans] than in shrews, yet the thickness of the cortex is only six times greater”. Like Rakic^44^, White et al^45^ emphasize the computational utility of expanding surface area over thickness within the radial unit lineage model that allows for “an optimized compaction of neuronal fibers with an efficient transit time for neuronal signaling”. Consequently, increased cortical gray matter with advancing decades of birth results in increased surface area through increased gyrification. According to the radial unit lineage model, the increased gyrification (and consequent surface area) leads to subtle reductions in cortical thickness as seen in our data. Importantly, both Rakic^44^ and White et al^45^ emphasized regional differences in “gyrification” are likely under unique genetic influence^46^. Such an analysis is beyond the scope of this report but could add further information regarding the biology of cortical gray matter expansion with subtle reduction in cortical thickness as seen with our more global measures.

Memory performance also improved with decade of birth and was significantly associated with all MRI measures except cortical thickness. Improved memory performance was dramatically associated with level of educational achievement, being nearly 1/3 of a standard deviation higher for college educated participants. Memory performance was nearly linearly associated with decade of birth for women but appeared to asymptote somewhat for men (Fig. 6). This too may be associated with achieved level of education for men versus women. Whether this relationship is due to increasing memory performance that leads to increased likelihood of college education or visa a versa is beyond the scope of this report and requires further investigation. Despite these sex differences, improvement in memory performance was partially mediated by increased hippocampal volume that also occurred with advancing decades of birth.

How might these secular effects modify the likelihood of later life dementia? Brain growth begins in utero, increases throughout childhood, and reaches a maximum size in early adulthood,^47–50^. TCV is highly associated with brain growth during normal development^40^, whereas aging or disease-related brain-volume decrease does not alter TCV. Thus, adult TCV is a stable valid measure for maximal attained brain size, widely used as a proxy for brain reserve^10, 51^, and is an important predictor of cognition in old age^41^ including studies that used similar measures of memory performance^51^. Conversely, cross-sectional, and longitudinal age-related differences in brain volume measures associate with cognitive performance in aging and disease^52–54^. Hippocampal volume loss, in particular, is considered to be sensitive to early degenerative diseases such as Alzheimer’s disease^55, 56^. Although absolute volumes do not associate with cognitive ability per se, as illustrated by the fact that women in this study had significantly smaller hippocampal volumes, but significantly better memory performance, loss of brain tissue within an individual is strongly indicative of pathological effects^57^, which, therefore, may be buffered by larger structural brain development. As noted in our data, while decade of birth had a Significant positive effect of cortical gray matter volume, the impact of aging remains essentially unchanged. Consequently, individuals in each decade of birth appear to atrophy at the same rate, but later decades start further away from any ‘threshold’ of atrophy that might manifest as clinical cognitive impairment. Alternatively, and more likely, larger structural brain development may be a surrogate for other environmental processes ongoing during development and early adult life such as increased brain connectivity^58^. Increased connectivity is consistent with the radial unit lineage hypothesis^44^ that enables increased neuronal connectivity through cortical expansion and gyrification^45^. Such increased connectivity could mitigate the impact of age-related diseases on cognitive performance and fits well with the scaffolding hypothesis of cognitive reserve^59^. Evidence supporting the notion that experience is associated with regional brain expansion can be seen among London taxi-cab drivers who have larger hippocampal volumes compared to same aged individuals^60^.

While head and brain size are under substantial genetic influence^61,46, 62–64^, the timeline of effect found with our results indicate that early life environmental influences are also likely substantial contributors, particularly educational achievement^6^. Life course perspectives emphasize the impact of early life experiences on brain health^65, 66^ that also translate into larger brain structures^67^ and reduced risk for later-life dementia through improved reserve^68^. Similarly, effort to improve cardiovascular health during adulthood^69–71^ are associated with reduced incidence of cognitive impairment^72^ and dementia^73^ indicating that modifying these factors could also serve to improve resistance to late-life dementia^74^.

In summary, our results indicate that TCV and brain structures have increased over birth years ranging from 1902 to 1985. These differences are coincident and associated with improved memory performance that is partially mediated by larger hippocampal volumes. These findings likely reflect both secular improvements in early life environmental influences through health, social cultural, and educational factors^67^ as well as secular improvements in modifiable dementia risk factors leading to better “brain health” and reserve^32^. While these effects are likely to be small at the level of the individual, they are likely to be substantial at the population level adding to growing literature that suggests optimized brain development and ideal health through modification of risk factors could substantially modify the impact of common neurodegenerative diseases such as stroke and Alzheimer’s disease on dementia incidence^6, 34, 70^. Moreover, taken together with increases in IQ throughout the 20th century^75^, these secular trends in brain volume may contribute to an overall more cognitively resilient and productive society.

The strengths of our study include the design of the Framingham Heart Study that began in 1948 and has followed a community of individuals with comprehensive health evaluations throughout much of their lifespan and across three generations. The addition of MRI beginning in 1999 enabled quantitative brain assessment across all three generations spanning more than 80 years difference in dates of birth. Obtaining contemporaneous MRI with neuropsychological assessment also enabled brain behavior associations. A high rate of participant participation resulting in a large sample size across decades of birth also enabled reliable sensitivity analyses that might not be accomplished with smaller studies. The duration of observation that includes younger individuals also suggests that this secular trend may be continuing. Finally, the fact that more than 80% of subjects were imaged on just two MRI machines also helped to reduce machine differences that were further reduced using the NeuroCombat statistical harmonization method.

This study, however, is not without limitations. First, and most importantly, the Framingham Heart Study is predominately non-Hispanic White, healthy, and well educated and, therefore, not representative of the more diverse US population. For example, current evidence indicates social-cultural^67, 76–78^ and health disparities^2^ which are more common among non-White individuals^32^ in the US may adversely affect brain health. Second, this is a cross-sectional study that has limited causal inference. Longitudinal analyses showing secular differences in rates of regional brain atrophy would further support evidence of increased brain reserve through resilience to age-related atrophy^39^. Similarly, we are not aware of another cohort spanning 3 or more decades of birth to validate these findings.

Despite these limitations, we conclude that this study extends current knowledge by showing that secular trends in brain structure are occurring and are associated with improved memory performance. We hypothesize that the larger brain structure reflects improved brain health and is at least one manifestation of improved brain reserve that could buffer the impact of late life diseases on incident dementia. Our results also support the growing literature that emphasizes optimal brain development and ideal brain health as preventive measures to mitigate against rising dementia prevalence among our aging US population.

## Figures and Tables

**Table 1:** Sample Characteristics * obtainment of a college degree (yes or no).

Variable	Men	Women	P value
Number (%)	2088 (46)	2418 (54)	
Age at MRI (years)	57 ± 12	57 ± 13	0.71
Year of Birth	1949 ± 16	1948 ± 16	0.48
College Educated* (%)	77	75	0.15
ApoE4 Prevalence (%)	22.5	22.3	0.92
Current Smoker (%)	11	10	0.47
Height at first visit (inches)	69.7 ± 2.6	64.2 ± 2.4	< 0.0001
Intracranial Volume (cc)	1356 ± 109	1200 ± 98	< 0.0001
Cortical Gray Volume (cc)	522 ± 48	468 ± 43	< 0.0001
Hippocampal Volume (cc)	7.05 ± 0.72	6.41 ± 0.64	< 0.0001
Cortical Thickness (mm)	2.11 ± 0.17	2.11 ± 0.17	0.47
Immediate Memory (number of words)	11.5 ± 3.4	12.5 ± 3.5	< 0.0001
Delayed Memory (number of words)	10.5 ± 3.5	11.6 ± 3.7	< 0.0001
FSRP (%10 year risk)	0.04 ± 0.06	0.03 ± 0.07	< 0.0001
Systolic Blood Pressure (mmHg)	124.4 ± 16	120 ± 19	< 0.0001
Diastolic Blood Pressure (mmHg)	76.0 ± 9.3	71.5 ± 9.4	< 0.0001
Hypertension Prevalence (%)	38	31	< 0.0001
Hypertension Treatment (%)	29	23	< 0.0001
Diabetes (%)	10.3	5.2	< 0.0001
History of Heart Disease (%)	11.3	6.9	< 0.0001

**Table 2:** Impact of Decade of Birth on Brain Structure

Predictors	MRI Variables^1^											
	Intracranial Volume			Cortical Gray			Hippocampus			Cortical Thickness		
Age group (years)	Total	45–75	55–65	Total	45–75	55–65	Total	45–75	55–65	Total	45–75	55–65
N	4506	3335	1259	4506	3335	1259	4506	3335	1259	4506	3335	1259
Age (years)	0.004	0.004	0.003	−0.021***	−0.02	−0.019*	−0.009**	−0.005	−0.012	−0.03***	−0.028***	−0.025*
Sex (Female)	−0.60***	−0.60***	−0.61***	−0.51***	−0.51***	−0.52***	−0.42 ***	−0.43***	−0.42***	0.016	−0.004	−0.013
Birth Decade	0.014***	0.016***	0.019***	0.008***	0.011***	0.014***	0.010***	0.012***	0.015***	−0.016***	−0.015***	−0.012*
Age × Birth Decade	0.000*	0.000	0.001	0.000	0.000	0.001	0.001***	0.001	0.002	0.000	0.000*	0.001
Sex × Birth Decade	−0.003	−0.002	0.004	−0.003	−0.002	0.001	−0.002	−0.001	0.002	0.005	0.004	0.003
Age × Sex	−0.002	−0.003	0.013	0.000	−0.001	0.013	0.001	−0.000	0.005	0.004	0.004	0.018
Age × Sex × Birth Decade	0.000	−0.000	−0.000	0.000	−0.000	−0.000	−0.000*	−0.000	0.001	0.000	−0.000	0.000
Model Adjusted R Squared	0.39***	0.39***	0.41***	0.43***	0.37**	0.35***	0.29***	0.25***	0.24***	0.04***	0.02***	0.01***

P<0.0001 ***,

<0.001 **

<0.05 *

**Table 3:** Impact of Decade of Birth on Memory Test Performance

Predictors	Memory Tests		
	Immediate	Delayed	Immediate+Delayed
Age at Memory Test	0.04	−0.04	0.001
Sex (Female)	0.15***	0.13***	0.15***
Decade of Birth	0.25***	0.17**	0.21***
Education (Coll)	0.26***	0.28***	0.28***
Decade of Birth × Age at Memory Test	0.04*	0.03*	0.04*
Model Adjusted R-squared	0.13***	0.14***	0.14***

P < 0.0001 ***,

< 0.001 **

<0.05 *

Memory tasks were z-transformed for comparison across test types. All interactions were included in the model, but only Significant interactions are presented here. Results are presented as beta coefficients from regression analyses.

**Table 4:** Causal Mediation Analysis of Hippocampus on Relation Between Decade of Birth and Memory Performance

Mediation Components	Immediate Memory	Delayed Memory	Combined
ACME	0.012 [0.005–0.02] ***	0.013 [0.006–0.02] ***	0.013 [0.006–0.02] ***
ADE	0.210 [0.123–0.29] ***	0.164 [0.080–0.25] ***	0.192 [0.108–0.28] ***
Total Effect	0.222 [0.138–0.31] ***	0.177 [0.094–0.26] ***	0.206 [0.121–0.29] ***
Proportion Mediated	0.054 [0.024–0.11] ***	0.073 [0.031–0.16] ***	0.063 [0.028–0.13] ***

*** p < 0.0001, [] = 95% confidence limits based on 5,000 bootstrap iterations

ACME denotes Average Causal Mediation Effects and ADE denotes Average Direct Effects.
